# TCR stimulation strength is inversely associated with establishment of functional brain-resident memory CD8 T cells during persistent viral infection

**DOI:** 10.1371/journal.ppat.1006318

**Published:** 2017-04-14

**Authors:** Saumya Maru, Ge Jin, Todd D. Schell, Aron E. Lukacher

**Affiliations:** Department of Microbiology and Immunology, Penn State College of Medicine, Hershey, Pennsylvania, United States of America; Brown University, UNITED STATES

## Abstract

Establishing functional tissue-resident memory (T_RM_) cells at sites of infection is a newfound objective of T cell vaccine design. To directly assess the impact of antigen stimulation strength on memory CD8 T cell formation and function during a persistent viral infection, we created a library of mouse polyomavirus (MuPyV) variants with substitutions in a subdominant CD8 T cell epitope that exhibit a broad range of efficiency in stimulating TCR transgenic CD8 T cells. By altering a subdominant epitope in a nonstructural viral protein and monitoring memory differentiation of donor monoclonal CD8 T cells in immunocompetent mice, we circumvented potentially confounding changes in viral infection levels, virus-associated inflammation, size of the immunodominant virus-specific CD8 T cell response, and shifts in TCR affinity that may accompany temporal recruitment of endogenous polyclonal cells. Using this strategy, we found that antigen stimulation strength was inversely associated with the function of memory CD8 T cells during a persistent viral infection. We further show that CD8 T_RM_ cells recruited to the brain following systemic infection with viruses expressing epitopes with suboptimal stimulation strength respond more efficiently to challenge CNS infection with virus expressing cognate antigen. These data demonstrate that the strength of antigenic stimulation during recruitment of CD8 T cells influences the functional integrity of T_RM_ cells in a persistent viral infection.

## Author summary

Tissue-resident memory (T_RM_) cells are a subset of memory T cells that primarily reside in non-lymphoid tissues and serve as sentinels and effectors against secondary infections. T_RM_ cells have been extensively characterized in mucosal barriers, but much less is known about this population in non-barrier sites such as the brain. In this study, we designed a novel strategy to evaluate the impact of T cell stimulation strength on the generation and functionality of memory CD8 T cells in both lymphoid and nonlymphoid tissues. Using a mouse polyomavirus (MuPyV) library expressing variants of a subdominant epitope recognized by TCR transgenic CD8 T cells, we found that systemic infection producing weaker responses during T cell priming was sufficient for recruitment of effector cells to the brain. Furthermore, lower stimulation conferred greater functionality to memory T cells in the spleen and to brain T_RM_ cells. Our findings demonstrate that the strength of antigenic stimulation experienced by a naïve T cell early in infection is a determinant of memory functional integrity during viral persistence in a non-barrier organ.

## Introduction

Following TCR engagement, pathogen-specific naïve CD8 T cells rapidly expand to generate a large effector population to counter primary infection, with a small population of memory CD8 T cells concomitantly generated to provide accelerated immunity to re-infection. CD8 T cell activation and differentiation requires three signals: TCR stimulation (signal 1), co-stimulation (signal 2), and inflammatory cytokines (signal 3), with the duration and intensity of these signals determining whether an activated CD8 T cell is fated towards an effector or memory state [1–5]. The canonical naïve-to-effector/memory differentiation profile for CD8 T cell responses to microbial infections is derived from analyzing T cell responses in secondary lymphoid organs. Tissue-resident memory (T_RM_) cells apparently circumvent this differentiation schema by “locking” themselves in an effector-poised state having a transcription profile distinct from circulating central-memory and effector-memory T cells [6–10].

Most studies to date have characterized T_RM_ cells in mucosal tissue barriers (e.g., skin, lung, gut, and female reproductive tract), where they act to provide rapid protection against secondary infections [11–16]. Less is known about the factors involved in establishment of T_RM_ cells in non-barrier organs, particularly the CNS, an organ system susceptible to irreparable injury by viral infection. Several viral CNS infection mouse models have described the establishment of T_RM_ cells in the brain. VSV encephalitis generates antiviral CD8 T_RM_ cells that persist in the brain long after antigen clearance [9, 17]. Using intracerebral (i.c.) inoculation with lymphocytic choriomeningitis virus (LCMV), Steinbach et al. showed that virus-specific CD8 brain-T_RM_ cells, in the absence of circulating antiviral CD8 T cells, conferred protection to CNS challenge with LCMV [12].

A variety of approaches have been devised to selectively define the role of TCR stimulation in T cell effector and memory differentiation. Antigen abundance has been controlled using mutant viruses that variably express a given T cell epitope, using low-dose inocula, and by limiting the duration of infection with antiviral agents [18, 19]. Genetic disruption of proximal TCR signal transduction has also been used to isolate the strength of signal 1 [20–23]. These studies have typically assessed the impact of such manipulations on dominant CD8 T cell responses to acutely cleared infections in secondary lymphoid organs. Whether changes in TCR stimulation affect the generation or functionality of T_RM_ cells remains to be determined.

In this study, we conceived a strategy to directly assess the impact of TCR stimulation strength on memory T cell formation. First, we focused on a subdominant CD8 T cell response to avoid confounding effects on viral replication and virus-associated inflammation that may result from alterations in a dominant T cell compartment. Second, we studied the differentiation of TCR transgenic donor CD8 T cells to control the size, timing of recruitment, and clonality of the naïve T cell compartment. Third, we used mouse polyomavirus (MuPyV), a natural mouse pathogen that establishes a persistent infection well controlled by the host immune system. Fourth, we altered an epitope in a nonstructural viral protein to avoid potential effects on host cell tropism that may occur with mutations in a capsid protein [24]. Using a library of MuPyVs with signal 1-altering mutations in a subdominant CD8 T cell epitope, we found that reduced TCR stimulation quantitatively and qualitatively improved the generation of memory T cells in both a lymphoid (i.e., spleen) and a nonlymphoid (i.e., brain) organ.

## Results

### Analogue epitopes differentially stimulate subdominant MuPyV-specific CD8 T cells

MuPyV-infected B6 mice generate a CD8 T cell response to three epitopes in the nonstructural viral T antigens, according to the following immunodominance hierarchy: D^b^-restricted Large T antigen (LT) amino acids 359–368 (LT359) > K^b^-restricted Middle T antigen (MT) amino acids 246–253 (MT246) > D^b^-restricted LT amino acids 638–646 (LT638) [25]. The D^b^LT359 and D^b^LT638 MuPyV epitopes share homology with SV40 LT epitopes in B6 mice; e.g., mutagenizing 4 of 10 codons in LT359 to match its homologous sequence in SV40 LT (residues 206–215) yielded an MuPyV mutant virus expressing a dominant epitope recognized by monoclonal SV40 LT-specific CD8 T cells [26].

**Fig 1:**
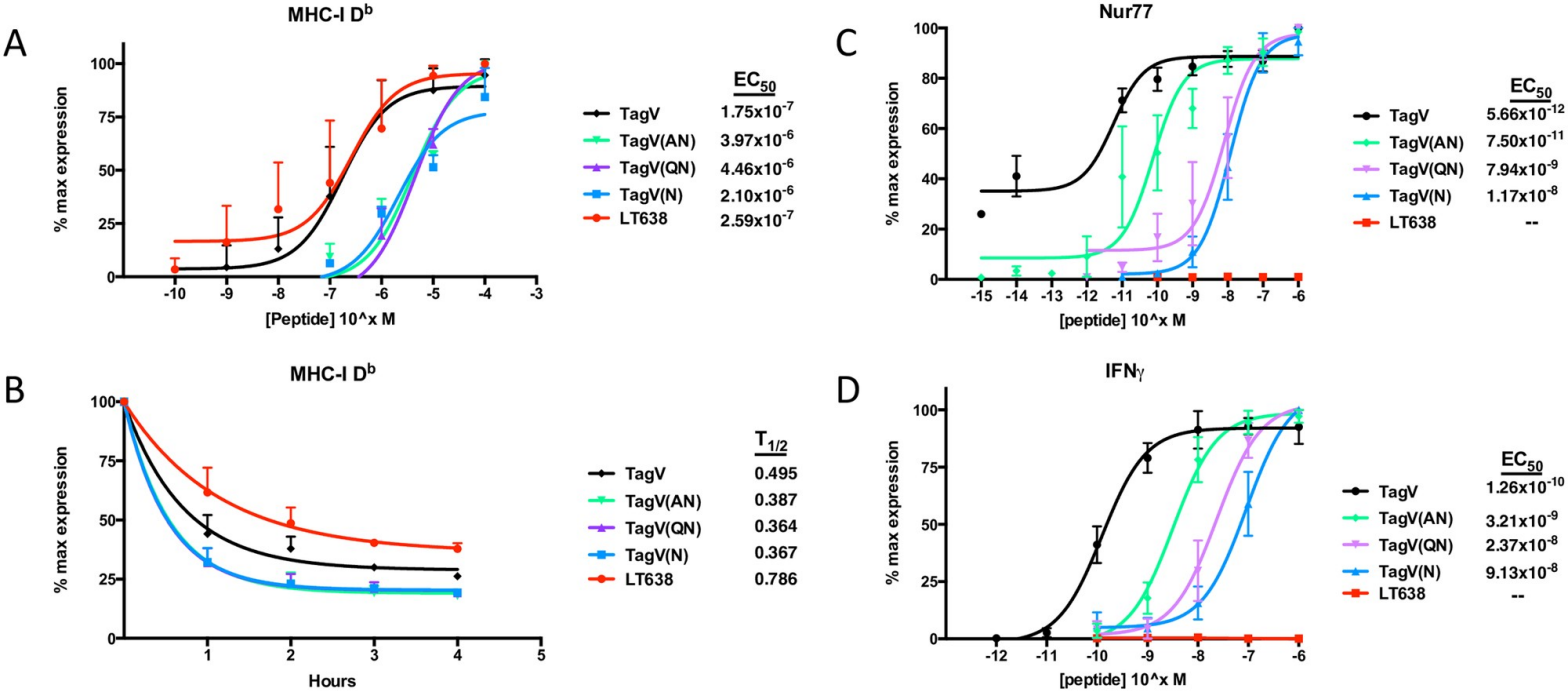
Characterizing the T cell response to analogues of a subdominant CD8 T cell epitope. (A) RMA/S cells were cultured overnight at 26°C. Cells were incubated with the indicated dose of peptide for 1 h at RT followed by incubation at 37C for 2 h. Table shows the EC_50_ of each analogue peptide for MHC-I binding and stabilization. (B) RMA/S cells were cultured overnight at 25°C in 100 μM of peptide. Cells were then resuspended in fresh media, incubated at 37C, and sampled hourly for 4 h. Table shows the half-life of the analogue peptides for binding to MHC-I. (C-D) Splenocytes were isolated from a naïve TCR-V mouse and stimulated with TagV analogue peptides at the indicated concentrations for 5 h at 37°C. Nuclear Nur77 (C) and intracellular IFNγ expression (D) shown. Table shows the EC_50_ of each analogue peptide for expression of these activation markers. All data plotted as percent of maximal expression ± SD.

Using this approach, we sought to develop a strategy to incisively assess the impact of antigen stimulation strength on the generation of CD8 memory T cells to MuPyV infection that avoided altering the kinetics and magnitude of infection. The subdominant MuPyV LT638 epitope and the “immunorecessive” SV40 LT epitope corresponding to amino acids 489–497, designated TagV, differ by three residues in the amino-half of their epitopes (Table 1). We previously demonstrated that replacing the P1 Gln of TagV with Ala improved stimulation of TCR-V transgenic CD8 T cells specific for the TagV epitope [27]. Thus, we constructed the following nonameric peptides that we predicted would differentially stimulate TCR-V cells: the native TagV and LT638 epitopes; and three variant LT638 epitopes where the P4 Ala was replaced with Asn (TagV(N)) either alone, or in combination with substitution of the P1 Met residue with Gln (TagV(QN)) or Ala (TagV(AN)) (Table 1). Using the RMA/S peptide stabilization assay, we determined that the native TagV and LT638 peptides efficiently bound D^b^ at equilibrium, but that the TagV dissociated from D^b^ faster than LT638 (Fig 1A and 1B). Each of the analogue peptides exhibited markedly weaker binding to and faster dissociation from D^b^. It is notable that these analogue peptides differentially associated with D^b^ despite retaining the dominant D^b^ anchors Asn at P5 and Leu at P9 [28].

**Table 1:** TagV analogues.

Peptide	Sequence	Virus	Primers
TagV	**Q**G**IN**NLDNL	MuPyV.TagV	F: CAG CCA GGG CAG GGA ATC AAT AAT CT R: GAG ATT ATC TAG ATT ATT GAT TCC CTG
TagV(AN)	**A**GV**N**NLDNL	MuPyV.TagV(AN)	F: CAG CCA GGG GCG GGA GTG AAT AAT CT R: GAG ATT ATC TAG ATT ATT CAC TCC CGC
TagV(QN)	**Q**GV**N**NLDNL	MuPyV.TagV(QN)	F: CAG CCA GGG CAG GGA GTG AAT AAT CT R: GAG ATT ATC TAG ATT ATT CAC TCC CTG
TagV(N)	MGV**N**NLDNL	MuPyV.TagV(N)	F: CAG CCA GGG ATG GGA GTG AAT AAT CT R: GAG ATT ATC TAG ATT ATT CAC TCC CAT
LT638	MGVANLDNL	MuPyV	—

Subdominant D^b^-restricted CD8 T cell epitopes of SV40 and MuPyV differ by three amino acids; analogue peptide sequences shown. Residues P5 Asn and P9 Leu are the dominant D^b^ anchor residues. Bold letters indicate residue changes made in the MuPyV LT638 epitope. Primer sets used to mutate residues to create the analogue TagV epitopes in parental MuPyV.

We next compared these peptides for their efficiency in stimulating naïve TCR-V CD8 T cells. Using a range of peptide concentrations, we assayed the ability of these peptides to induce production of IFNγ, a cytokine typically used to assess T cell “functional avidity”, as well as Nur77, an orphan nuclear receptor that is a direct measure of TCR signaling strength [29–31]. As shown in Fig 1C and 1D, TCR-V cells recognized the native TagV peptide but not the LT638 epitope from MuPyV, with intracellular Nur77 staining showing approximately 1-log higher sensitivity than intracellular IFNγ for detecting antigenic stimulation. Interestingly, both IFNγ and Nur77 readouts revealed a fine gradient of antigenic stimulation efficiency by the analogue peptides as follows: TagV > TagV(AN) > TagV(QN) > TagV(N). Notably, the three analogue peptides differ in TCR stimulation and D^b^ binding; e.g., the TagV(AN) and TagV(N) peptides exhibit comparable binding to and dissociation from D^b^ (Fig 1A & 1B), but the former is approximately 2 logs more efficient in stimulating TCR-V cells. Collectively, these data indicate that these substitutions concomitantly affect MHC and TCR binding and provide a set of analogue peptides to interrogate the impact of antigen stimulation strength on T cell differentiation.

### Suboptimal TCR stimulation improves recruitment of MuPyV-specific CD8 T cells

**Fig 2:**
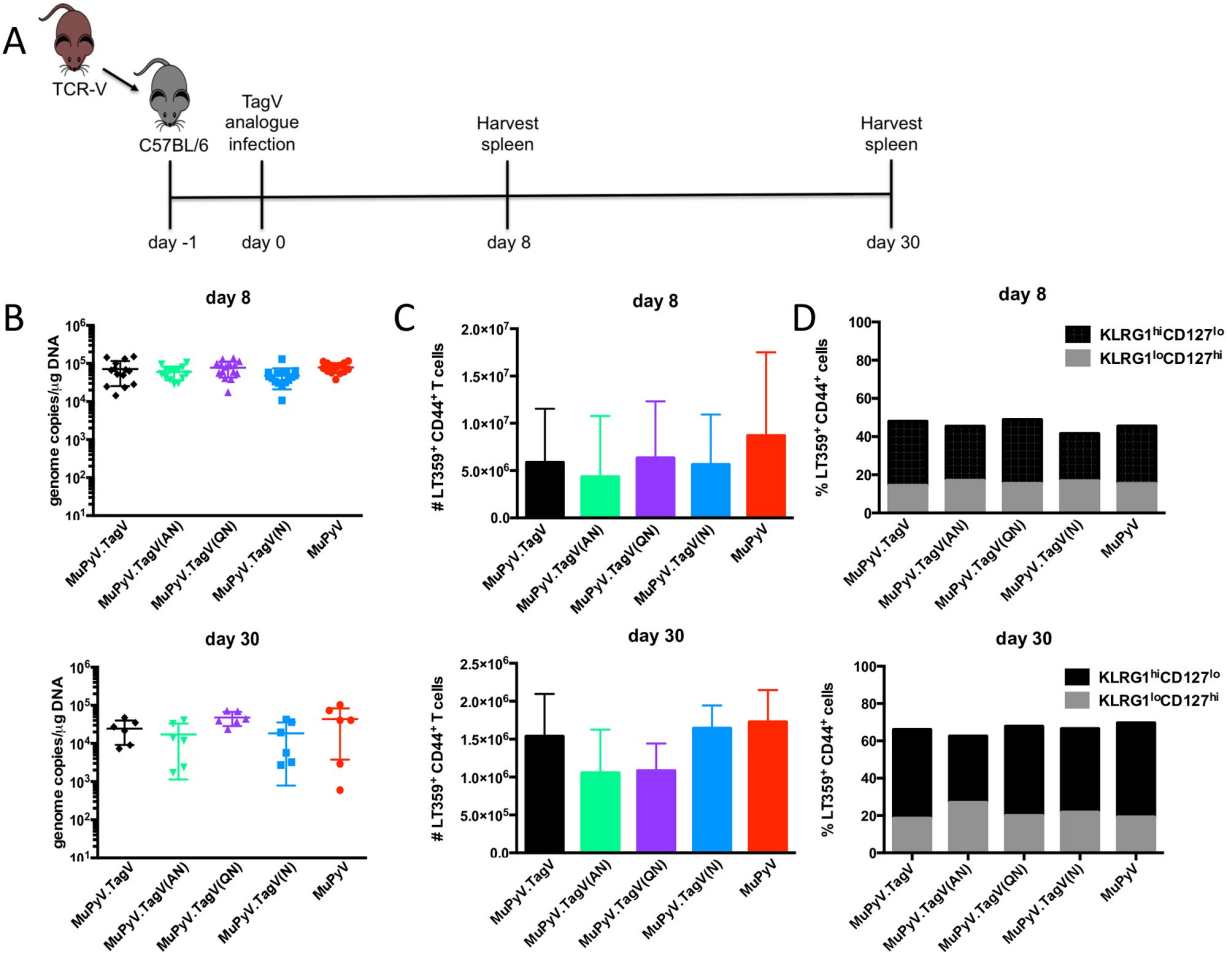
Infection by analogues of the subdominant TagV epitope does not affect the endogenous anti-MuPyV response. (A) 1 x 10^3^ TCR-V CD8 T cells were adoptively transferred i.v. into B6 mice. Mice were infected with 2 x 10^6^ PFU of MuPyV via hind footpads the following day and sacrificed at day 8 or day 30 p.i. (B) Viral load in the spleen at day 8 (top panel) or day 30 (bottom panel) p.i. (C) Number of LT359-specific CD8 T cells in the spleen at day 8 p.i. (top panel) or day 30 p.i. (bottom panel). (D) Percent of LT359-specific CD8 T cells that are KLRG1^hi^CD127^lo^ (black) and KLRG1^lo^CD127^hi^ (gray) at day 8 (top panel) or day 30 (bottom panel) p.i. Mean ± SD plotted: no significant differences. N = 12–18 mice from 4–6 independent experiments.

Using site-directed mutagenesis of the MuPyV genomic DNA, we altered the coding sequence of the native LT638 epitope to that of TagV and to each of the three TagV analogue peptides, and isolated infectious virus. As depicted in Fig 2A, our approach involved adoptive transfer of a “physiologic” number (1,000) of naïve TCR-V CD8 T cells into CD45-congenically disparate B6 mice which were then inoculated with parental MuPyV or one of the four MuPyV variants carrying mutations in the LT638 epitope. We first assessed whether this experimental design altered the dynamics of acute (day 8 p.i.) and persistent (day 30 p.i.) MuPyV infection as well as the generation and maintenance of the CD8 T cell response to the dominant D^b^LT359 epitope. As shown in Fig 2B and 2C, the mutant viruses were indistinguishable from parental MuPyV in their replicative efficiency in vivo and recruitment of the dominant D^b^LT359-specific CD8 T cell anti-MuPyV response. Furthermore, effector-memory differentiation of the D^b^LT359 population based on the KLRG1 and IL-7Rα (CD127) co-expression paradigm [32, 33] revealed no differences between parental or analogue MuPyV infection (Fig 2D).

**Fig 3:**
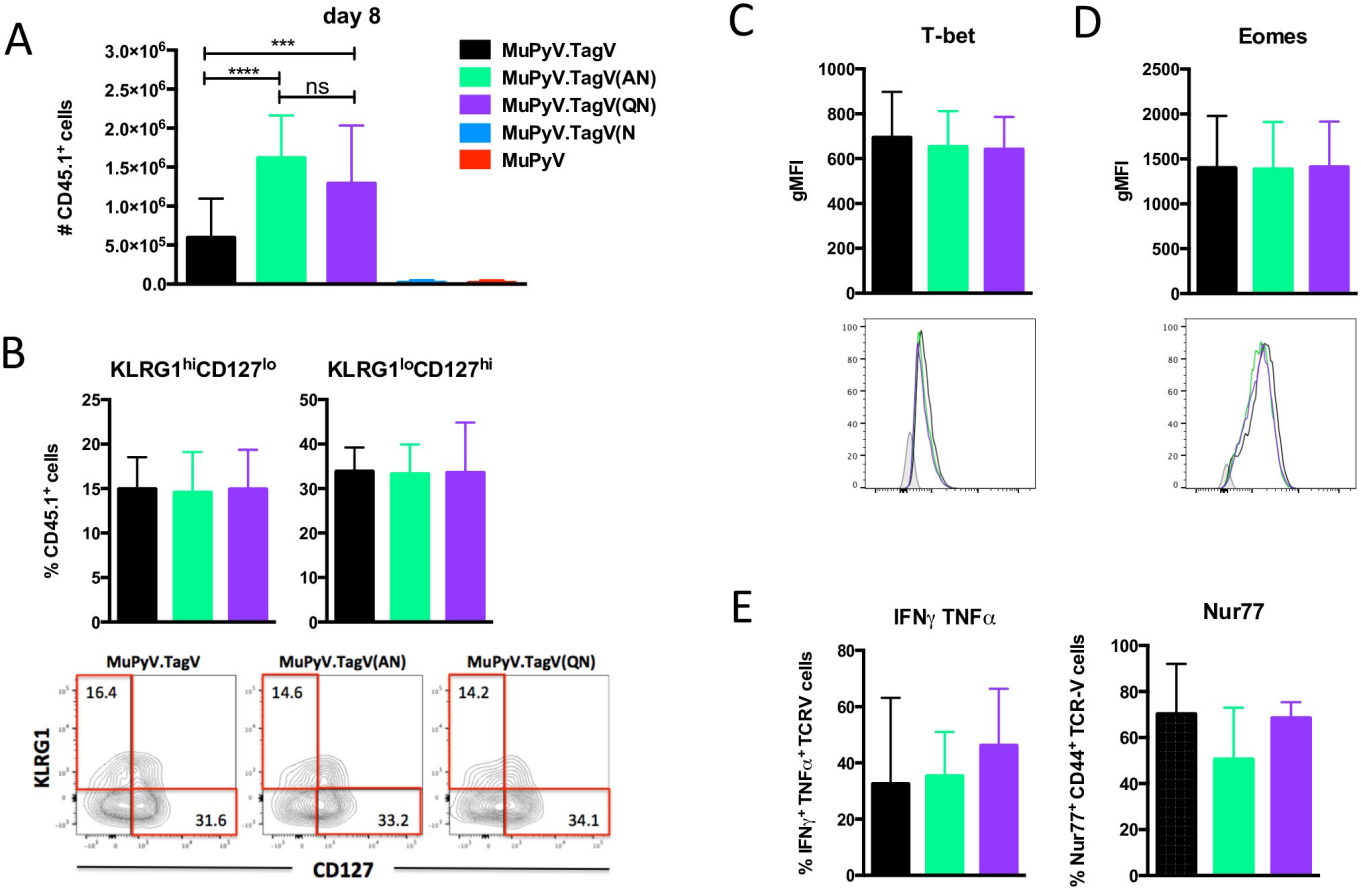
TCR stimulation strength does not affect CD8 T cell memory differentiation in the spleen during acute infection. TCR-V cells were transferred i.v. into B6 mice one day prior to infection with TagV analogue viruses and sacrificed at day 8 p.i. (A) Number of TCR-V cells in the spleen. (B) Percent of TCR-V cells that are KLRG1^hi^CD127^lo^ (left panel) or KLRG1^lo^CD127^hi^ (right panel). Representative dot plots shown. (C-D) Expression of T-bet (C) and eomes (D) by splenic TCR-V cells. Representative histograms shown. Gray shaded histograms represent fluorescence-minus-one controls. (E) IFNγ and TNFα co-expression (left panel) and Nur77 expression (right panel) in splenocytes stimulated for 5 h ex vivo with 1 μM TagV peptide. Mean ± SD plotted. N = 9–18 mice from 3–6 independent experiments.

Infection with the analogue viruses uncovered marked differences in efficiency in recruitment of TCR-V CD8 T cells. During the acute phase of infection, TCR-V cells mounted the largest response to infection by mutant viruses carrying the TagV(AN) and TagV(QN) epitopes, which significantly exceeded the magnitude of the response to infection by MuPyV.TagV, which expresses the cognate TCR-V epitope (Fig 3A). TCR-V cells were not detected in spleens of mice infected with parental MuPyV (as expected) or the mutant TagV(N) virus carrying the single P4 Ala-to-Asn substitution in the LT638 epitope; thus, all subsequent experiments used only the TagV, TagV(AN), and TagV(QN) variant viruses. Based on KLRG1 and CD127 co-expression and expression of the T-box transcription factors T-bet and eomesodermin (eomes), no differences were seen in markers of effector and memory differentiation by TCR-V cells recruited by MuPyV.TagV, MuPyV.TagV(AN), and MuPyV.TagV(QN) infection (Fig 3B–3D). Moreover, direct ex vivo stimulation with cognate TagV peptide induced similar expression of Nur77 and IFNγ/TNFα co-functionality by TCR-V cells (Fig 3E) despite differences between these three viruses in their capacity to stimulate naïve TCR-V cells (Fig 1C & 1D).

### Lower TCR stimulation increases memory CD8 T cell function

**Fig 4:**
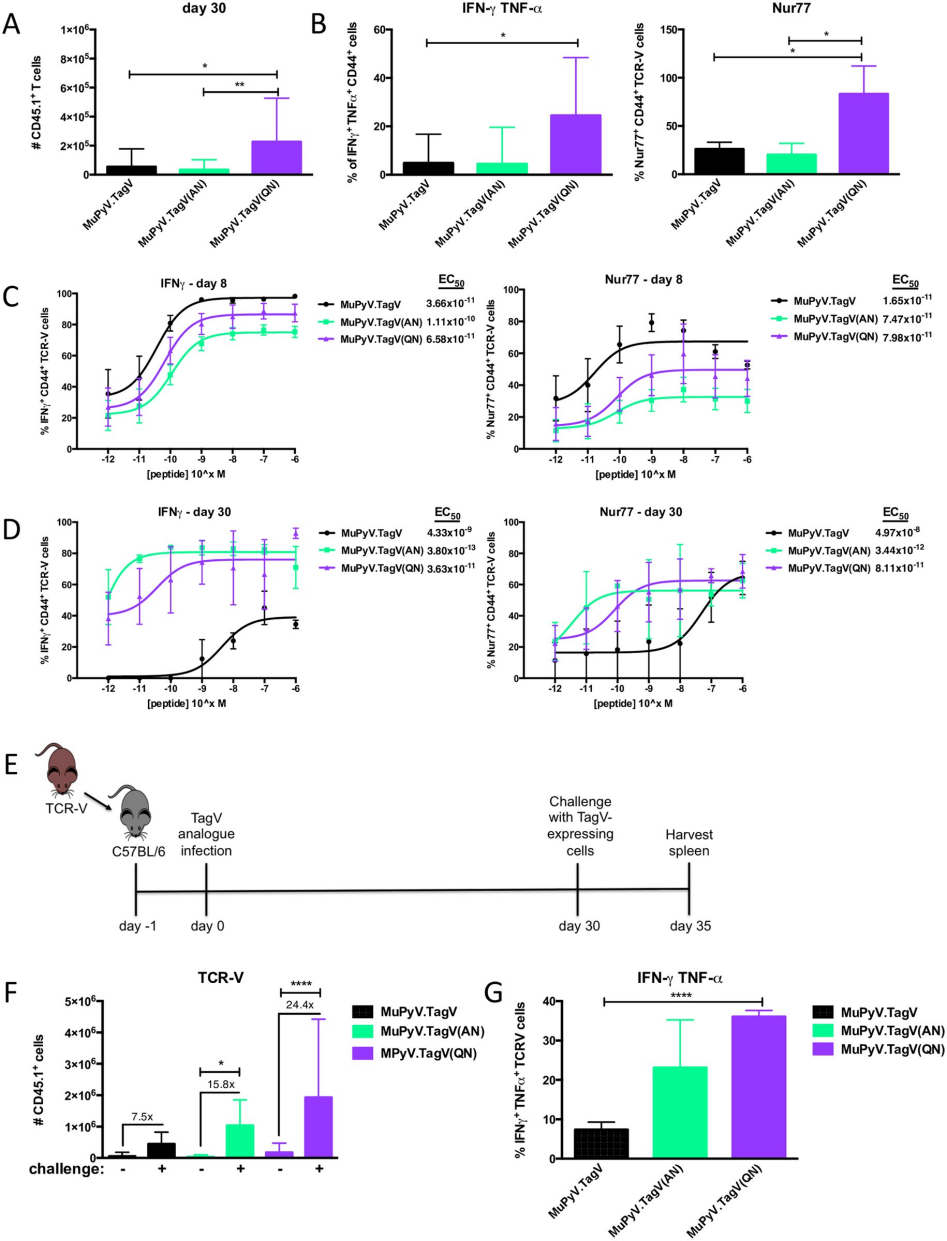
Memory splenic CD8 T cells primed with lower TCR stimulation exhibit superior recall responses. (A-B) TCR-V cells were adoptively transferred into B6 mice one day prior to infection with cognate Tag V or TagV analogue viruses, then sacrificed at day 30 p.i. (A) Number of TCR-V cells in the spleen. (B) IFNγ and TNFα co-expression (left panel) and Nur77 expression (right panel) in splenocytes stimulated for 5 h ex vivo with 1 μM TagV peptide. (C-D) Intracellular IFNγ (left panel) and Nur77 (right panel) expression by TCR-V cells isolated from the spleen at day 8 p.i. (C) or day 30 p.i. (D) stimulated for 5 h ex vivo with varying concentrations of the cognate TagV peptide. (E) 1 x 10^3^ donor TCR-V CD8 T cells were transferred into B6 mice. Mice were infected with 2 x 10^6^ PFU of MuPyV via hind footpads the following day, challenged i.p. with 5 x 10^7^ 116A1 cells at day 30 p.i., and sacrificed five days post-challenge. (F) Number of TCR-V cells in the spleen with or without secondary challenge and fold change of TCR-V cells in mice receiving secondary challenge compared to day 30 averages. (G) IFNγ and TNFα co-expression in splenocytes stimulated for 5 h ex vivo with 1 μM TagV peptide. Mean ± SD plotted. N = 6–18 mice from multiple independent experiments. *, p < 0.05; ****, p < 0.0001; ANOVA with Tukey’s test for significance.

Because strong, sustained antigen stimulation dampens the functionality of CD8 T cells, we next asked whether lower TCR stimulation could improve memory T cell differentiation. At day 30 p.i., TCR-V cells recruited in response to infection by TagV, TagV(AN), and TagV(QN) mutant MuPyVs gave rise to a population of memory T cells, with MuPyV.TagV(QN) infection generating a significantly larger population (Fig 4A). When stimulated ex vivo with 1μM cognate TagV antigen, TCR-V cells primed by MuPyV.TagV(QN) infection yielded memory cells that were capable of co-producing IFNγ and TNFα to a significantly higher level than those recruited by MuPyV.TagV or MuPyV.TagV(AN) infection (Fig 4B, left panel). Notably, a significantly higher fraction of TagV(QN) epitope-primed memory CD8 T cells produced Nur77 as well, indicating that these T cells possessed improved sensitivity to the cognate antigen (Fig 4B, right panel). To address this possibility, we performed peptide dose-response curves on TCR-V effector and memory cells from the spleen. Splenocytes from day 8- and day 30-infected mice were stimulated for five hours with TagV, TagV(AN), TagV(QN), TagV(N), or LT638 peptide at concentrations ranging from 1 μM to 1 pM, then analyzed for intracellular IFNγ and Nur77. Effector and memory TCR-V cells exhibited higher functional avidity to the cognate TagV peptide than to the analogue peptides irrespective of which of these viruses was used for the primary infection, suggesting that a recall response would be optimally elicited by cognate antigen (S1 Fig). Effector TCR-V cells from MuPyV.TagV-infected mice stimulated with cognate TagV peptide had a lower EC_50_ for IFNγ and Nur77 expression than those from MuPyV.TagV(AN)- and MuPyV.TagV(QN)-infected mice (Fig 4C). By day 30 post infection, however, memory TCR-V cells primed with MuPyV.TagV(AN) and MuPyV.TagV(QN) infection acquired higher functional avidity, a three-to-four log decrease in EC_50_, than those recruited with MuPyV.TagV infection (Fig 4D), indicating an evolution toward lower activation threshold for cognate antigen for memory cells primed with a weaker TCR stimulus.

We next compared the in vivo antigen recall responses of TCR-V memory cells generated by infection to TagV, TagV(AN), and TagV(QN) mutant MuPyVs. Following systemic infection with MuPyV, mice mount a robust neutralizing antibody response that prevents repeat infection with the same virus. Thus, we used TagV epitope-expressing fibroblasts, 116A1 cells, to present a new source of TagV antigen [34]. 116A1 cells were injected intraperitoneally (i.p.) at day 30 p.i., and splenic TCR-V cells were enumerated five days later (Fig 4E). Both MuPyV.TagV(AN) and MuPyV.TagV(QN) infected mice showed a significant increase in TCR-V cell expansion upon 116A1 cell challenge compared to infection-matched unchallenged mice, with TagV(QN)-primed TCR-V cells exhibiting the highest level of expansion (Fig 4F). When stimulated ex vivo with cognate TagV antigen, TagV(QN)-primed secondary effector TCR-V cells had significantly improved co-production of IFNγ and TNFα (Fig 4G). Together, these data show a marked increase in memory potential in TCR-V cells primed with lower TCR stimulation.

### Systemic MuPyV infection recruits tissue-resident memory CD8 T cells to the brain

**Fig 5:**
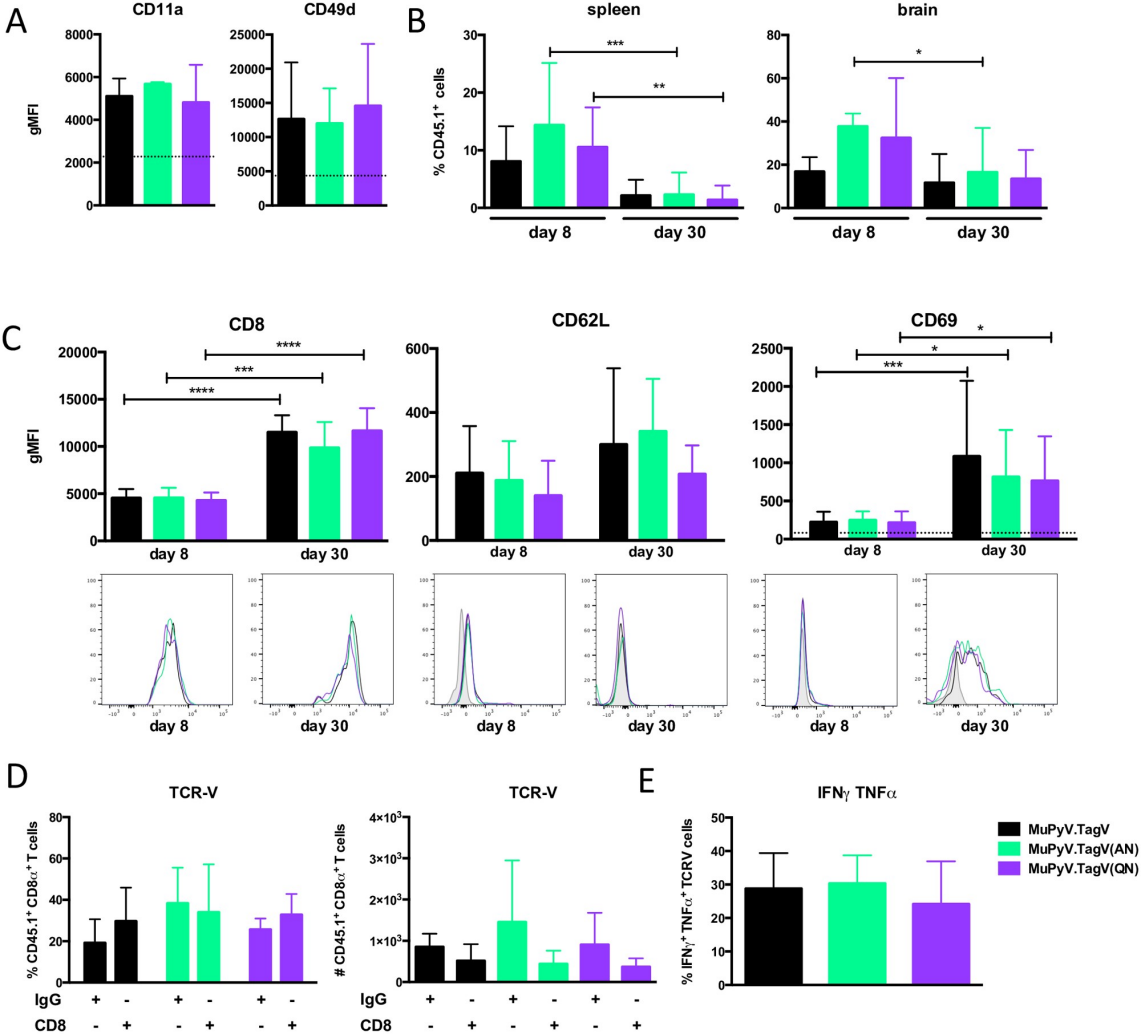
Systemic infection with TagV analogue MuPyVs recruits anti-PyV CD8 T cells to the brain. (A) Circulating TCR-V cells at day 8 p.i. show increased expression of adhesion markers CD11a and CD49d. Dashed line indicates baseline expression on naïve TCR-V cells. (B) Percentage of TCR-V cells in the spleen (left panel) and brain (right panel) at days 8 and 30 p.i. (C) gMFI of CD8, CD62L, and CD69 expression on TCR-V cells in the brain at days 8 and 30 p.i. Representative histograms shown. Gray shaded histogram refers to fluorescence-minus-one control. (D) Percentage (right panel) and number (left panel) of TCR-V cells in the brains of mice given systemic anti-CD8α or control rat IgG. (E) IFNγ and TNFα co-expression in brain-resident TCR-V cells stimulated for 5 h ex vivo with 1μM TagV peptide. Mean ± SD plotted. N = 9–15 mice from 3–5 independent experiments. *, p < 0.05; **, p, 0.005; ***, p< 0.0005; ANOVA with Tukey’s test for significance.

T_RM_ cells protect against microbial infections in non-lymphoid tissues [11–16]. Because JC polyomavirus causes a devastating demyelinating brain disease [35], we asked whether differential TCR signaling from systemic infection affected establishment of brain-resident TCR-V memory cells. CD11a and CD49d, subunits of the LFA-1 and VLA-4 integrins, respectively, involved in CNS entry by circulating T cells [36, 37], were upregulated in circulating TCR-V cells at day 8 p.i. irrespective of infection with MuPyV.TagV, Tag(AN), or Tag(QN) viruses. (Fig 5A). As shown in Fig 5B, the TCR-V cell population responding to infection by viruses with the cognate TagV epitope and the TagV(AN) and TagV(QN) analogue epitopes underwent expansion and contraction in the spleen, and contracted after migrating into the brain but to a lesser extent than in the spleen. Compared to splenic TCR-V cells (S2A Fig) and brain-infiltrating TCR-V cells at day 8 p.i., brain-derived memory TCR-V cells for each virus expressed low CD62L, elevated CD69, as well as elevated CD8α (Fig 5C), a phenotype consistent with T_RM_ cell differentiation [6]. CD103 expression was not detected on these cells (S2B Fig), a finding in line with evidence from us and others that CD103 is not a faithful T_RM_ marker [38]. Further confirmation that these are T_RM_ cells comes from the finding that their presence in the brain was unaffected following depletion of circulating CD8 T cells (Figs 5D & S3). Brain-resident TCR-V cells generated in response to infection by TagV, TagV(AN), or TagV(QN) MuPyVs exhibited similar cytokine effector capability upon stimulation by the cognate TagV peptide (Fig 5E). Together, these data indicate that lower TCR stimulation in the context of a systemic viral infection generates functional brain-T_RM_ cells.

### Brain-resident memory cells primed with lower stimulation have improved memory function

**Fig 6:**
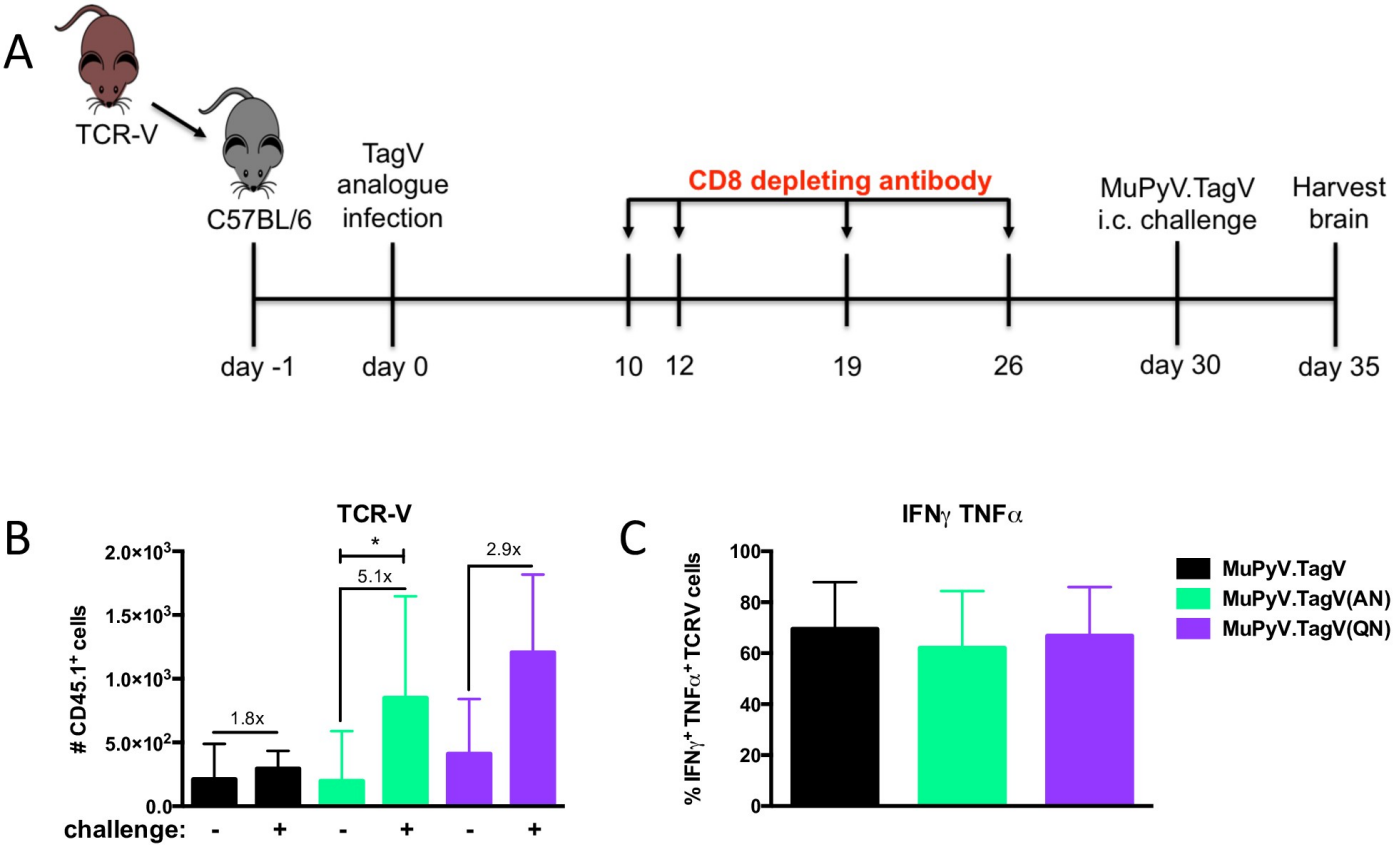
Brain-T_RM_ cells primed with lower stimulation exhibit increased recall potential. (A) 1 x 10^3^ donor TCR-V CD8 T cells were adoptively transferred into recipient C57BL/6 mice. Mice were infected with 2 x 10^6^ PFU of MuPyV via hind footpads the following day and given depleting anti-CD8α on day 10 p.i. and once/week for 3 weeks. Mice were challenged i.c. with MuPyV.TagV virus on day 30 p.i. and sacrificed five days post-challenge. (B) Number of TCR-V cells in the brain with or without secondary challenge (left panel) and fold change of TCR-V cells in mice receiving secondary challenge compared to day 30 averages (right panel). (C) IFNγ and TNFα co-expression from TCR-V cells stimulated for 5 h ex vivo with 1 μM TagV peptide. Mean ± SD plotted. N = 6–15 mice from 2–5 independent experiments. *, p < 0.05; ANOVA with Tukey’s test for significance.

We next compared the recall responses by brain T_RM_ cells recruited by infection with MuPyV.TagV, MuPyV.TagV(AN), and MuPyV.TagV(QN) viruses. A strongly neutralizing antibody response in MuPyV-immune mice negates the ability to rechallenge mice systemically with MuPyV. Because the blood-brain barrier largely excludes circulating immunoglobulins, we investigated the possibility that i.c. injection of MuPyV would allow reinfection in the brain in MuPyV-immune mice. On day 30 p.i., mice were challenged i.c. with MuPyV.TagV virus and sacrificed five days later (Fig 6A). To confine analysis to brain-TCR-V T_RM_ cells, mice received CD8 depleting mAb starting day 10 p.i. after the cells had migrated to the brain. We observed that only TCR-V T_RM_ cells generated by MuPyV.TagV(AN) infection underwent significant expansion to MuPyV.TagV challenge infection (Fig 6B). Despite these differences in size of the recall response, no significant differences in cytokine effector capability were seen (Fig 6C). Together, these data demonstrate that infection with MuPyV mutants carrying a suboptimally stimulating epitope can promote establishment of functional T_RM_ cells having an improved capacity to counter re-infection.

## Discussion

In this study, we show that strength of TCR stimulation is a central determinant guiding the differentiation of functional antiviral CD8 T_RM_ cells in persistent infection. To insulate effects of TCR stimulation from dynamic changes associated with host immunity, and do so in a natural host viral infection, we mutated a weak subdominant CD8 T cell epitope in MuPyV and monitored the differentiation of donor CD8 T cells from a TCR transgenic mouse. By altering a subdominant CD8 T cell epitope within a virus and not impacting viral fitness or tropism, we circumvented variations in antigen processing, virus-associated inflammation, and host antiviral immunity that could impact T cell fate. In addition, adoptive transfer of a physiologic number of TCR transgenic CD8 T cells served to eliminate evolution of the polyclonal response and timing of T cell recruitment on T cell differentiation [39, 40]. Using this strategy, we found that infection by MuPyVs carrying altered epitopes that reduced TCR stimulation strength (1) recruited a larger number of antiviral effectors than those elicited by cognate antigen, and (2) generated a memory population in both lymphoid and nonlymphoid tissues endowed with superior recall response capability. These findings provide direct evidence that TCR signal strength, as an isolated variable, plays a dominant role in the differentiation of T_RM_ cells.

A number of approaches have been used to investigate the influence of TCR signaling strength in T cell differentiation, including genetic alterations in TCR signaling, and modulating the level and duration of antigen and inflammation [18, 41, 42]. Inducible systems have been developed to disrupt proximal TCR signaling at the level of Lck [22], SLP-76 [23], and the TCR [20]. These studies collectively suggest that while TCR signaling is required for activation and expansion of naïve CD8 T cells, it is not required for memory T cell maintenance, self-renewal, or a secondary response. In contrast, OT-I cells with a point mutation in the TCR transmembrane domain that retains peptide:MHC ligand binding and proximal signaling, but has reduced NF-κB activity, undergo accelerated and deeper contraction and exhibit defective memory development [21]. Antigen availability has also been shown to affect the magnitude of CD8 T cell recruitment, but without impacting memory function, implicating an “all-or-nothing” phenomenon [19]. Temporal ablation of dendritic cells carrying a diphtheria toxin receptor transgene to vary the duration of antigen presentation curtailed the magnitude of the T cell response, but did not impede memory generation [43]. Other studies using antibiotic/antiviral treatment [32, 44] or transfer of early effector cells into infection-controlled hosts [33] to shorten the duration of antigen and inflammation have shown improvement in memory formation. We previously demonstrated that reducing the MuPyV inoculation dose was associated with increased memory T cell fitness [45]. It merits pointing out that each of these prior studies assessed T cell expansion and effector memory differentiation on a population level. At the single-cell level, TCR signaling appears to behave in a digital fashion, such that clonal expansion is engaged once a minimum threshold of TCR activation is reached [31]. Our finding that MuPyVs with altered subdominant epitopes drive equivalent or greater T cell clonal expansion than the native epitope fit with this concept; our data are also at odds with the notion of a positive correlation between TCR stimulation strength and clonal burst size.

Alteration of memory T cell differentiation as a function of TCR stimulation has been studied using analogue peptide epitopes that either differentially engage TCRs (altered peptide ligands; APLs) or MHC molecules (MHC variant peptides; MVPs). Using APLs of the K^b^-restricted SIINFEKL epitope with varying affinities for OT-1 cells expressed by recombinant *Listeria monocytogenes*, Zehn et al. found a direct correlation between affinity of the priming APL and OT-1 cell numbers. This correlation was explained by preferential retention of OT-1s in secondary lymphoid organs when priming involved the higher affinity APLs, such that cell numbers peaked earlier than with the lower affinity APLs; irrespective of APL affinity, OT-1 cells differentiated into functional memory cells [46]. In contrast, we found that weaker ligands for TCR-V cells elicited a larger expansion, and did so without affecting the kinetics of the response. This discrepancy may result from differences in innate signals induced by *L*. *monocytogenes* vs MuPyV infection, in acutely vs persistently infecting pathogens, the dominant K^b^/SIINFEKL OT-1 epitope vs the weak subdominant D^b^/TagV TCR-V epitope, and/or in the TCR stimulation strength of the analogue epitope ligands used in these infection models. Regardless of the MuPyV mutant used, TCR-V cells were not detected in the lymph nodes or the spleen prior to day 6 p.i. (S4 Fig). Furthermore, the relative percentage of TCR-V cells in the spleen at day 6 p.i. mirrored the trend seen at day 8 p.i., suggesting that the increased magnitude of TCR-V expansion in MuPyV.TagV(AN) infected mice was not due to earlier recruitment or delayed egress. Using an LCMV mutant carrying an MVP of the GP33 epitope recognized by P14 transgenic CD8 T cells, Evavold and colleagues observed early contraction of P14 cells due to accelerated apoptosis [47]. Although none of the analogue TagV peptides had changes in the dominant D^b^ anchoring residues (P5 asparagine and P9 leucine), these analogues reduced stabilization of D^b^ by RMA/S cells (Fig 1A); thus, the TagV analogues appear to straddle the line between APLs and MVPs. In any event, MuPyVs expressing analogue epitopes that have weaker TCR stimulatory capacity elicit a larger expansion of donor TCR-V cells than those carrying the cognate epitope (Fig 3).

A central finding in this study is that the strength of TCR stimulation affects the generation of CD8 T_RM_ cells. We found that lower TCR stimulation in the setting of a persistent viral infection generates brain T_RM_ cells with superior anamnestic potential and having increased functional avidity (Figs 4 & 6). It is possible that the increased functionality of TCR-V cells recruited by lower stimulation was due to unique programming. No differences in expression of T-bet/eomes among memory TCR-V cells generated in response to infection with the analogue viruses were detected (Fig 3); however, we cannot exclude the possibility for differences in epigenetics or other transcription factors shown to guide memory differentiation [48]. What we did observe, however, was an improvement in polyfunctionality and a dramatic increase in functional avidity of TCR-V cells primed with lower stimulation (Fig 4). While effector TCR-V cells recovered at day 8 p.i. from MuPyV.TagV-infected mice displayed slightly increased sensitivity to cognate TagV peptide, memory T cells recovered from MuPyV.TagV(AN)- and MuPyV.TagV(QN)-infected mice had superior functional avidity for cognate antigen, with EC_50_ values approximately three-to-four logs lower than the MuPyV.TagV-infected mice (Fig 4C & 4D). This result fits with the possibility that persistent low TCR stimulation, here via modulating residues in a cognate epitope, fosters generation of memory T cells having elevated functionality to the cognate epitope.

Functional avidity, an experimental readout reflective of TCR-pMHC affinity, TCR levels and topology, co-receptor levels, and number and activation of signaling molecules [49], has been shown to evolve with the state of differentiation of both polyclonal and TCR transgenic cells [50–52]. Indeed, our results show that memory TCR-V cells have increased functional avidity compared to naïve TCR-V cells (Figs 1, 4 & S1). Both TCR (CD3ε gMFI) and co-receptor (CD8α gMFI) levels were unchanged in either the spleen or the brain over the course of infection with MuPyV.TagV or the analogue viruses (S5 Fig). An increase in functionality of monoclonal populations of effector and memory cells, as seen with the TCR-V:MuPyV infection system, has been attributed to clustering of TCRs and signaling molecules to immunological synapses [53–55], and increased efficiency of signal transduction [50, 56].

Although the theme of lower TCR stimulation conferring greater memory potential holds true in both organs, we noted a disparity in the best responders to secondary challenge between the splenic memory T cells and the brain T_RM_ cells, in that cells generated to MuPyV.TagV(QN) infection exhibited higher recall responses in the spleen, but those in the brain were higher for memory TCR-V cells recruited by MuPyV.TagV(AN) infection (Figs 4 & 6). This difference might be explained by the different antigen challenge approaches (s.c. implantation of transformed TagV^+^ cells vs i.c. inoculation with TagV^+^ MuPyV). The superior recall response exhibited by the TagV(AN)- or TagV(QN)-primed TCR-V cells may in part also reflect the lower frequency of these cells at the time of secondary infection, which could extend the period of antigen availability and enable a longer secondary expansion phase.

Although the brain-resident CD8 T cells in our study lacked expression of CD103, a surface marker implicated in defining T_RM_ cell populations, it should be noted that CD103 appears to not be a reliable indicator for identifying T_RM_ cells [6, 57]. Expression of CD103 may be dependent on route of infection and the subsequent level of inflammation and recruitment of cells into the brain parenchyma, both of which are low in the brain following a systemic MuPyV infection. A defining characteristic of T_RM_ cells is their lack of dependence on replenishment from the circulation; indeed, depletion of CD8 T cells from the circulation did not affect stability of TCR-V cell numbers in the brain (Fig 5). Further studies are needed to assess the impact of route of inoculation and subsequent changes in local inflammation and antigen abundance on the generation of T_RM_ cells.

A sizeable body of literature indicates that subdominant CD8 T cells are an integral component of anti-microbial immunity. Studies in respiratory syncytial virus (RSV) and *Mycobacterium tuberculosis* have established a protective role for subdominant CD8 T cells, which in some cases have been shown to be more effective at clearing pathogens than the dominant T cell population [58, 59]. A study of HIV-1 vaccine design approached the idea of subdominant epitope vaccinations by inactivating immunodominant epitopes to foster compensatory expansion of the subdominant antiviral CD8 T cell populations, and showed that they were capable of inducing robust, protective responses [60]. A vaccine formulation that elicited multiple Ebola virus epitope-specific CD8 T cell populations, including those to subdominant epitopes, displayed significant protective capability [61].

T cell vaccine design typically focuses on generating high-affinity, dominant epitope-specific CD8 T cells. Weak TCR-antigen interactions, however, are capable of inducing expansion and phenotypic differentiation of naïve CD8 T cells into effector and memory cells [46, 62, 63]. Strong, sustained interaction of antigen with TCRs upregulates inhibitory receptors on CD8 T cells, rendering them dysfunctional [64–67]. Moreover, host-pathogen interactions are dynamic, leading to selection of pathogens with mutations in epitopes that interfere with recognition by pathogen-specific T cells [68–72]. Epitopes targeted by dominant T cell responses are especially susceptible to mutations that handicap binding to an MHC molecule and/or interfere with TCR engagement. Our data demonstrate that T_RM_ cells directed to a subdominant epitope are generated by infection with a persistent viral pathogen; these subdominant T_RM_ cells are endowed with strong memory potential such that they expand locally and produce antiviral cytokines during secondary challenge (Figs 4 & 6). By extension, evidence presented here highlights the importance of investing in vaccine strategies to generate subdominant memory T cell populations in nonlymphoid tissues.

In summary, we have explored the effects of TCR stimulation strength in a subdominant T cell population on the formation of lymphoid and brain-resident memory cells. Whereas clonal expansion and CD8 effector function have typically been considered to correlate directly with TCR stimulation, we have shown that lowering TCR stimulation strength results in larger T cell clonal expansion and maintains effector function while improving memory potential. Furthermore, our data provides evidence that systemic infection by a persistent virus expressing analogue epitopes with decreased TCR stimulatory capacity can recruit higher functioning tissue-resident memory cells to the brain.

## Materials and methods

### Ethics statement

All experiments involving mice were conducted with the approval of Institutional Animal Care and Use Committee (Protocols 45575 and 46194) of The Pennsylvania State University College of Medicine in accordance with the Guide for the Care and Use of Laboratory Animals of the National Institutes of Health. The Pennsylvania State University College of Medicine Animal Resource Program is accredited by the Association for Assessment and Accreditation of Laboratory Animal Care International (AAALAC). The Pennsylvania State University College of Medicine has an Animal Welfare Assurance on file with the National Institutes of Health’s Office of Laboratory Animal Welfare; the Assurance Number is A3045-01.

### Mice

Female C57BL/6 (B6) were purchased from the National Cancer Institute (Frederick, MD). CD45.1 TCR-V transgenic mice expressing a TCR specific for Large T antigen (LT) amino acids 498–505 (“SiteV”) of SV40 have been previously described [73]. Mice were used at 8–10 weeks of age.

### Cells

RMA/S cells [74] were cultured in RPMI 1640 medium supplemented with 10% FBS, 100 units/ml penicillin, and 100 μg/ml streptomycin. TagV-only expressing 116A1 cells (B6/T 116A1 Cl-C) [75], were cultured in DMEM supplemented with 10% FBS, 100 units/ml penicillin, 100 μg/ml streptomycin.

### Synthetic peptides

HPLC-purified peptides synthesized by Peptide 2.0, Inc. (Chantilly, VA) were dissolved in PBS and stored at -20°C. Peptides are listed in Table 1.

### Viruses

The A2 strain of MuPyV was prepared in baby mouse kidney cells as described [76]. TagV variant viruses were created using the QuikChange Site-Directed Mutagenesis kit (Agilent Technologies). Primer sets (Integrated DNA Technologies) are listed in Table 1. Viruses were titered by plaque assay on BALB/3T3 clone A31 cells (American Type Culture Collection, Manassas VA) as described [77]. Mice were infected in hind footpads with 2 x 10^6^ PFU.

### RMA/S peptide stabilization assays

For MHC-I stabilization assays, RMA/S cells were cultured overnight at 26°C, resuspended in RPMI containing 10% FBS (10% RPMI) and peptides of different concentrations, then incubated for 1 h at room temperature followed by 2 h at 37°C. For peptide dissociation assays, RMA/S cells were cultured overnight at 26°C in the presence of 100 μM peptide, then resuspended in 10% RPMI at 37°C. Each hour for 4 h an aliquot of cells was stained for 30 mins at 4°C with phycoerythrin-conjugated anti-D^b^ (clone 28-14-8; eBioscience), acquired on a BD FACSCanto10 instrument, and the geometric mean fluorescence intensity (gMFI) determined using FlowJo Software (Tree Star). The percent maximum gMFI was calculated as (gMFI^peptide^-gMFI^no peptide^)/(gMFI^max^- gMFI^no peptide^) x 100. EC_50_ and *T*_1/2_ were calculated using Prism software (GraphPad, La Jolla CA).

### Intracellular mAb staining

Splenocytes were cultured for 5 h in DMEM containing 10% FBS, 100 units/ml penicillin, 100 μg/ml streptomycin and supplemented with 1ug/ml brefeldin A (Sigma-Aldrich). Cells were exposed to Fixable Viability Dye eFluor 780 (eBioscience), then surface stained with mAbs to CD8α (clone 53–6.7; eBioscience), CD44 (clone IM7; eBioscience), and CD45.1 (clone A20; BioLegend), permeabilized with Cytofix/Cytoperm buffer (BD Biosciences) or FoxP3 buffer (eBioscience), and stained for intracellular IFNγ (clone XMG1.2; Biolegend), TNFα (clone TN3-19.12; eBioscience), IL-2 (clone JES6-5H4; BD Biosciences), and Nur77 (clone 12.14; eBioscience). D^b^LT359 and SiteV tetramers [25, 75] were provided by the NIH Tetramer Core Facility (Atlanta, GA).

### CD8 T cell isolation and adoptive cell transfer

CD8 T cells were purified from CD45.1 TCR-V mice using a negative selection CD8 T cell isolation kit (Miltenyi Biotec) according to manufacturer’s instructions; transferred cells were >90% CD8^+^. 1 x 10^3^ TCR-V cells were injected i.v. per tail vein one day prior to infection.

### Viral genome quantitation

TaqMan real-time PCR was performed using 10 μg template DNA purified from snap frozen tissues using the Maxwell 16 nucleic acid isolation system (Promega, Madison WI) as described [25].

### T cell isolation and flow cytometry

Spleens and brains were harvested at indicated days p.i. Brains were minced and digested with collagenase (100 U/ml in DMEM with 2% FBS, 200 U/ml penicillin, 200 g/ml streptomycin, 2 mM L-glutamine, 5 μM HEPES, 1 μM MgCl_2_, 1 μM CaCl_2_) for 30 min at 37°C, passed through a 70 μm nylon cell strainer (BD Biosciences). Cells were isolated by centrifugation on a 44%:66% Percoll gradient. Spleens were passed through a 70 μm nylon cell strainer, then treated with ACK buffer (0.15 M NH_4_Cl, 1mM KHCO_3_, 1mM Na_2_EDTA, pH 7.0) to lyse RBCs. Cells were surface-stained in FACS Buffer (PBS, pH 7.2 with 1% BSA, 0.1% sodium azide) for 30 min at 4°C with mAbs to CD8α (clone 53–6.7; Biolegend), CD44 (clone IM7; eBioscience), CD45.1 (clone A20; Biolegend), CD62L (clone MEL-14; BD Biosciences), CD69 (clone H1.2F3; BD Biosciences), PD-1 (clone RMP1-30; Biolegend), CD11a (clone 2D7; BD Biosciences), CD49d (clone MFR4.B; BioLegend), KLRG1 (clone 2F1; BD Biosciences), and CD127 (clone AFR34; Biolegend). Samples were collected on a BD LSR Fortessa or FACSCanto10 flow cytometer. Fluorescence-minus-one (FMO) samples were used to set positive gates for each surface molecule examined.

### CD8 T cell depletion

Mice were injected i.p. with 250 μg rat anti-CD8α (YTS169.4; Bio X Cell, West Lebanon NH) or ChromPure whole rat IgG (Jackson ImmunoResearch Laboratories, West Grove PA) at 10, 12, 19, and 26 days p.i. Depletion was confirmed in the blood by flow cytometry Absolute Count Standard (Bangs Laboratories, Fishers IN).

### TCR-V cell recall response

To assay splenic TCR-V recall, 5x10^7^ 116A1 cells in 0.5 ml PBS were injected i.p. To assay TCR-V recall in the brain, 2 x 10^6^ PFU MuPyV.TagV virus was injected i.c. in 30 μl DMEM containing 2% FBS. Mice were challenged at day 30 p.i. and sacrificed five days post-challenge.

### Statistical analysis

All data are displayed as mean ± SD unless otherwise indicated. *p* values were determined using an unpaired Student’s *t* test assuming equal variance or one-way ANOVA using GraphPad Prism software. All *p* values ≤ 0.05 were considered significant.

## Supporting information

S1 Fig Functional avidity of effector and memory TCR-V cells responding to infection by MuPyVs carrying cognate or analogue TagV epitopes. Intracellular IFNγ expression by TCR-V cells isolated from the spleen at day 8 (A) or day 30 p.i. (B) that were stimulated for 5 h ex vivo with varying concentrations of cognate or analogue TagV peptides.
(TIF)

S2 Fig Expression of CD8α, CD62L, CD69, and CD103 on effector and memory TCR-V cells. **(A)** gMFI of CD8α (left panel), CD62L (middle panel), and CD69 (right panel) on TCR-V cells in the spleen at days 8 and 30 p.i. with analogue viruses. (B) gMFI of CD103 on TCR-v cells in the spleen and brain at days 8 and 30 p.i. with analogue viruses.
(TIF)

S3 Fig Efficiency of antibody-mediated depletion of non-CNS CD8^+^ cells. Number of CD8 T cells pre-depletion (day 10 p.i.) and post-depletion (day 29 p.i.) with CD8^+^ cell-depleting antibody. CD8 T cells were depleted 42-fold in the blood (A), 13.2-fold in the spleen (B), and 8.7-fold in the cervical lymph nodes (C).
(TIF)

S4 Fig TCR-V cell expansion in the spleen and cervical lymph nodes. (A) Percent of TCR-V cells in the cervical lymph nodes at days 2, 5, 8, and 30 p.i. (B) Percent of TCR-V cells in the spleen at day 6 and day 8 p.i.
(TIF)

S5 Fig TCR and CD8 co-receptor expression on effector and memory TCR-V cells. gMFI of CD3 (A) and CD8 (B) on TCR-V cells from the spleen (right panels) and brain (left panels) at days 8 and 30 p.i.
(TIF)
