# Supplementary figures and images for: TCR stimulation strength is inversely associated with establishment of functional brain-resident memory CD8 T cells during persistent viral infection
Source: PLoS Pathog. 2017 Apr 14;13(4):e1006318. doi: 10.1371/journal.ppat.1006318 (PMC5406018; doi:10.1371/journal.ppat.1006318)

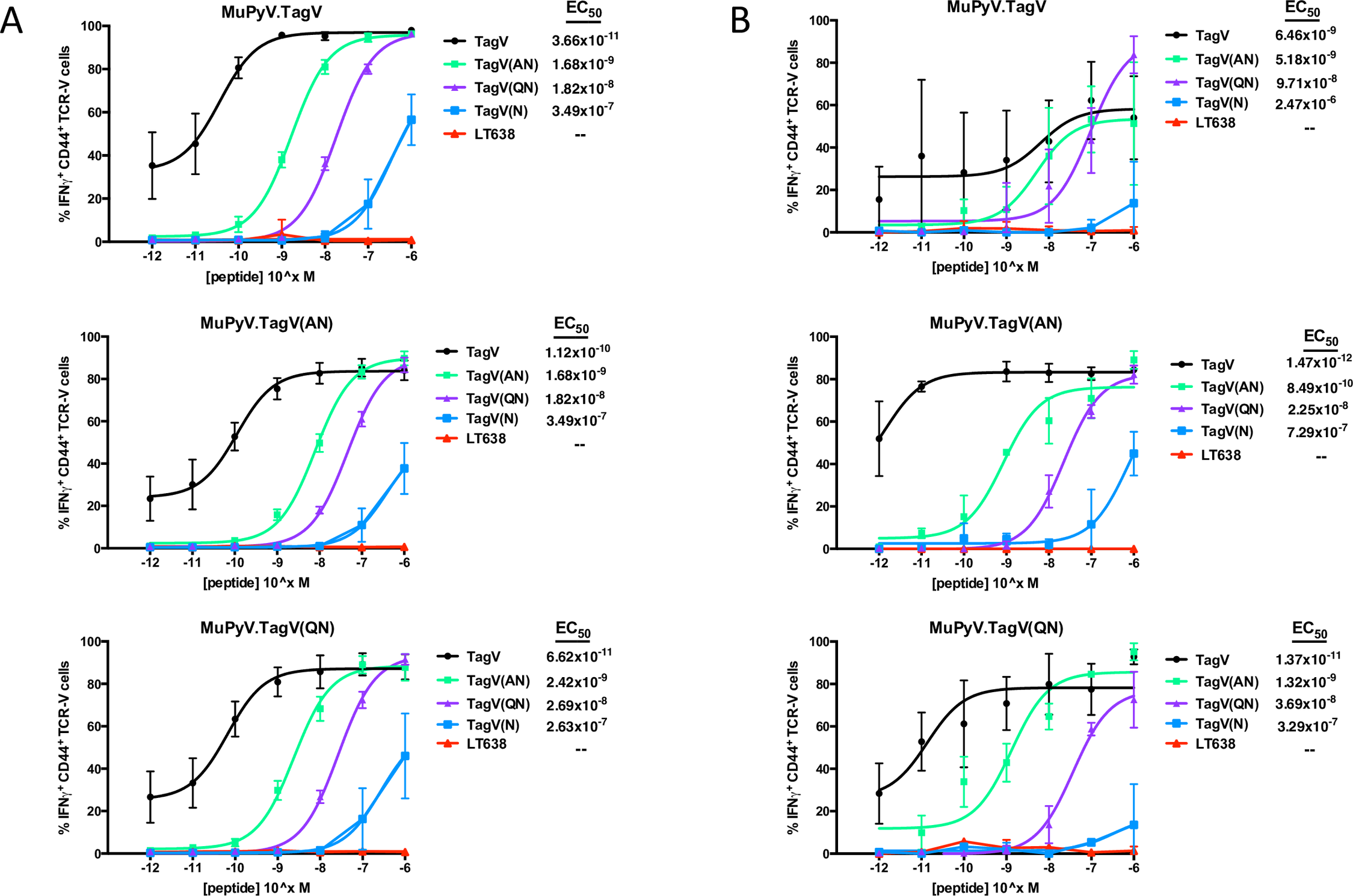

Supplement: S1 Fig — Intracellular IFNγ expression by TCR-V cells isolated from the spleen at day 8 (A) or day 30 p.i. (B) that were stimulated for 5 h ex vivo with varying concentrations of cognate or analogue TagV peptides. (TIF) [file ppat.1006318.s001.tif]

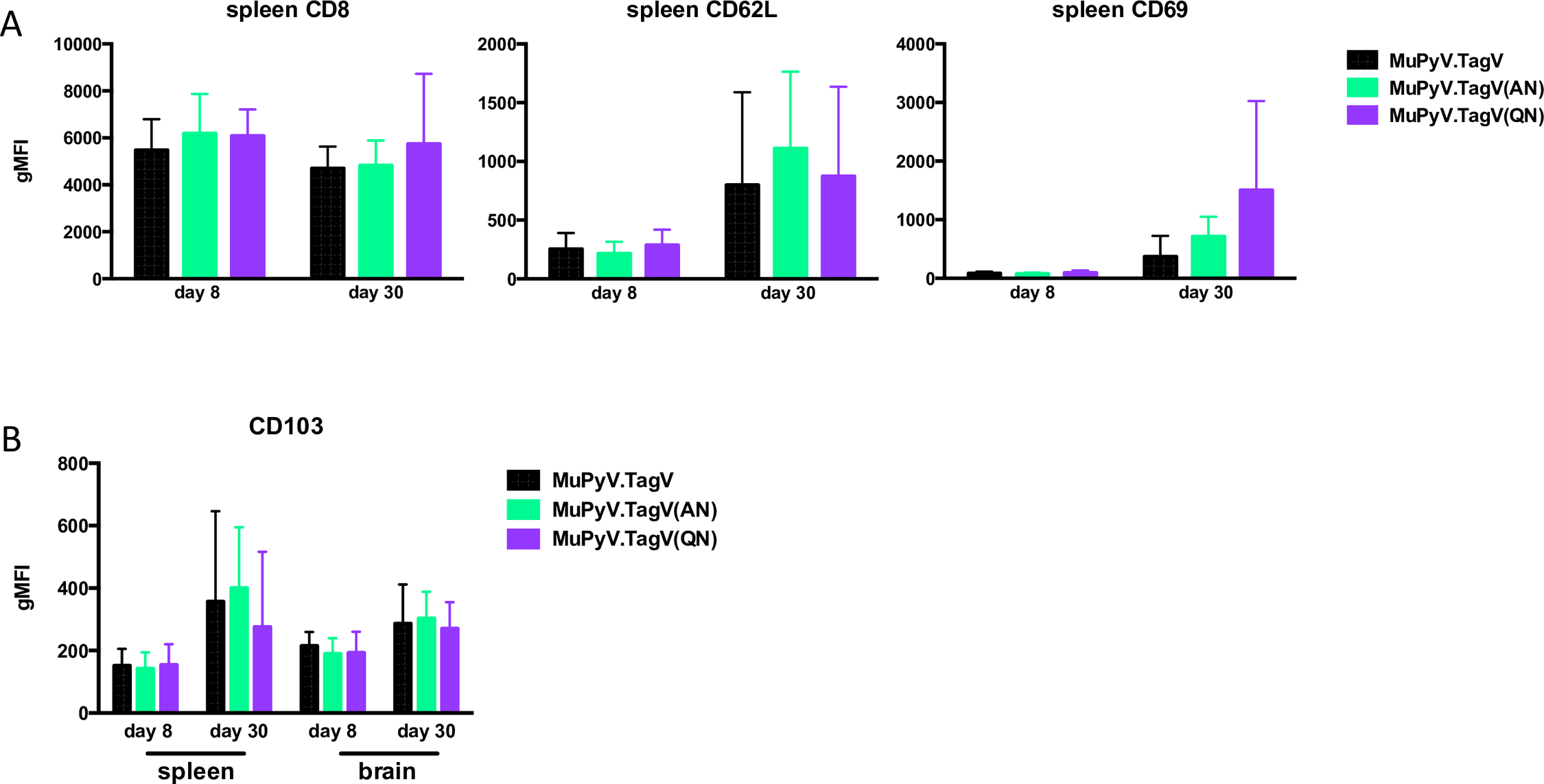

Supplement: S2 Fig — (A) gMFI of CD8α (left panel), CD62L (middle panel), and CD69 (right panel) on TCR-V cells in the spleen at days 8 and 30 p.i. with analogue viruses. (B) gMFI of CD103 on TCR-v cells in the spleen and brain at days 8 and 30 p.i. with analogue viruses. (TIF) [file ppat.1006318.s002.tif]

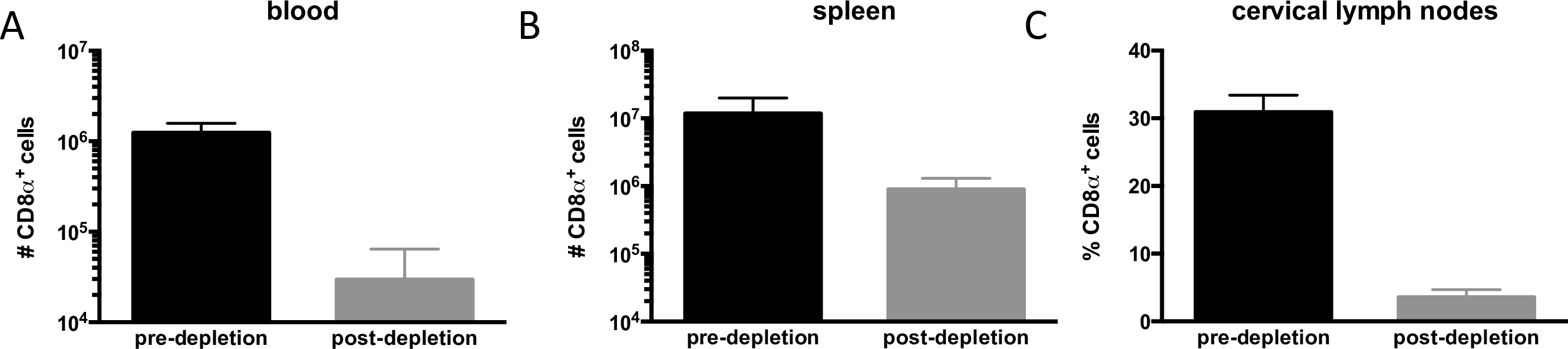

Supplement: S3 Fig — Number of CD8 T cells pre-depletion (day 10 p.i.) and post-depletion (day 29 p.i.) with CD8+ cell-depleting antibody. CD8 T cells were depleted 42-fold in the blood (A), 13.2-fold in the spleen (B), and 8.7-fold in the cervical lymph nodes (C). (TIF) [file ppat.1006318.s003.tif]

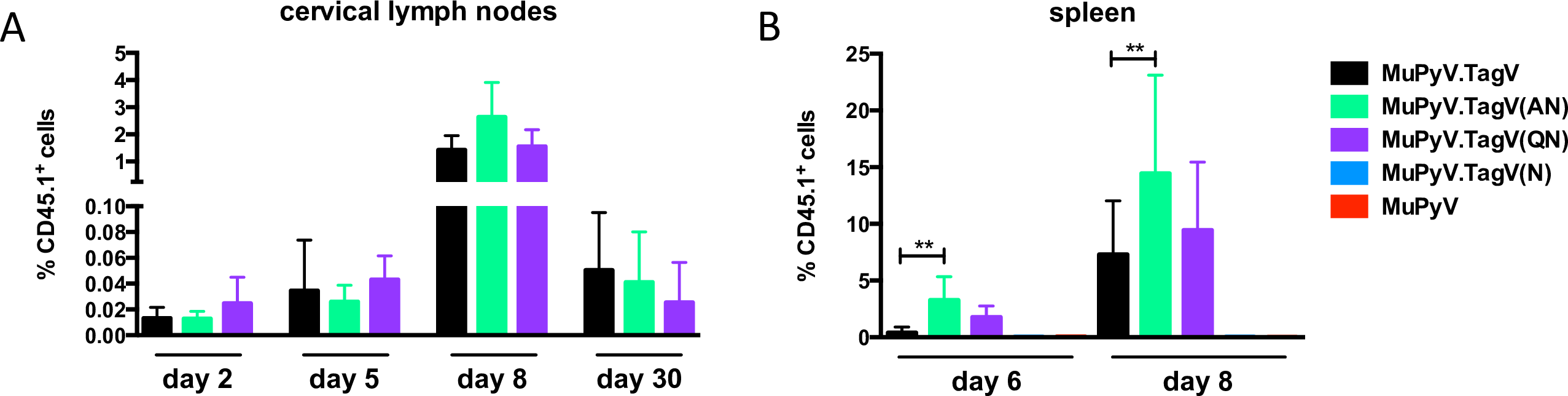

Supplement: S4 Fig — (A) Percent of TCR-V cells in the cervical lymph nodes at days 2, 5, 8, and 30 p.i. (B) Percent of TCR-V cells in the spleen at day 6 and day 8 p.i. (TIF) [file ppat.1006318.s004.tif]

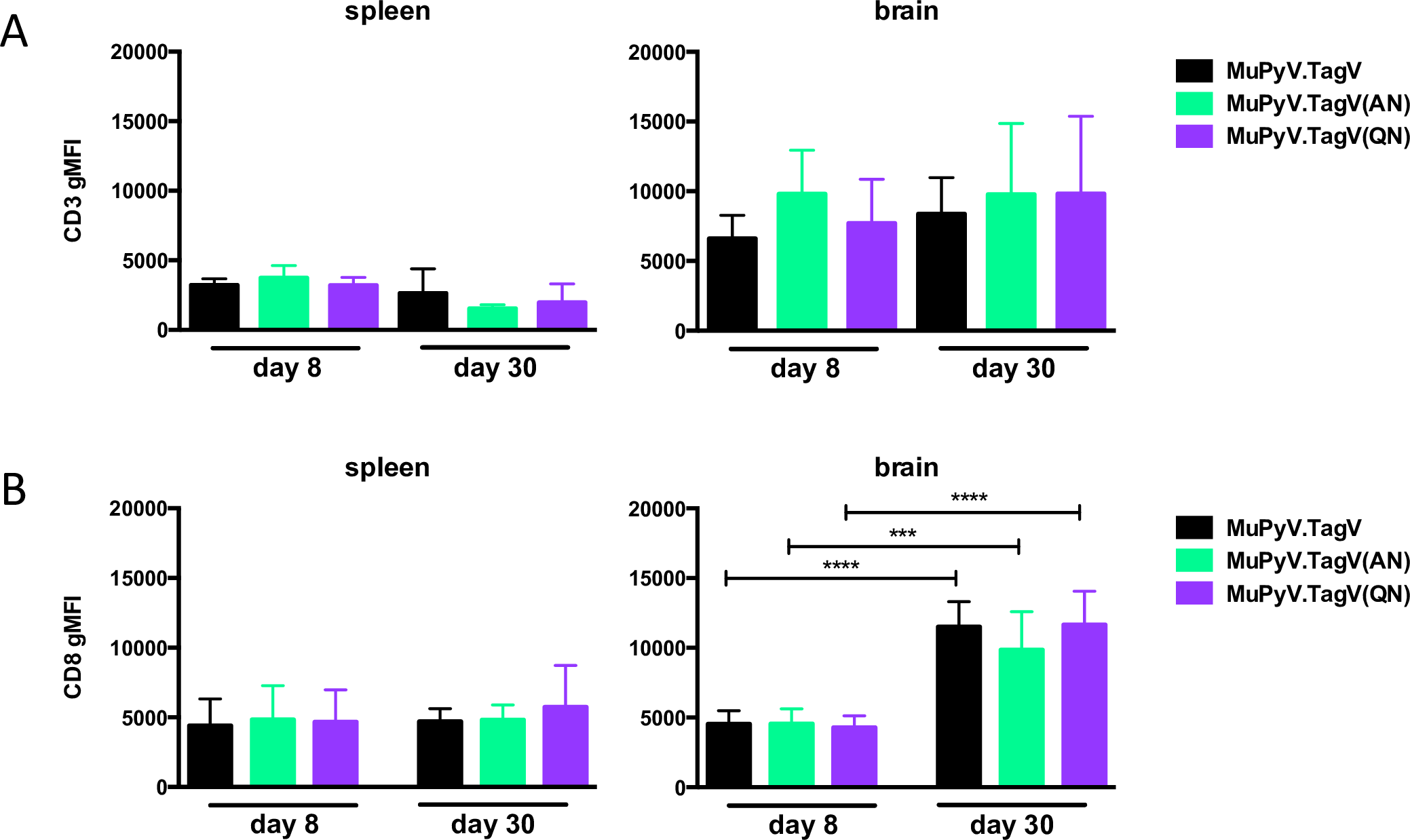

Supplement: S5 Fig — gMFI of CD3 (A) and CD8 (B) on TCR-V cells from the spleen (right panels) and brain (left panels) at days 8 and 30 p.i. (TIF) [file ppat.1006318.s005.tif]
